# Carotid Body AT_4_ Receptor Expression and its Upregulation in Chronic Hypoxia

**DOI:** 10.2174/1874192400701010001

**Published:** 2007-06-11

**Authors:** Man-Lung Fung, Siu-Yin Lam, Tung-Po Wong, Yung-Wui Tjong, Po-Sing Leung

**Affiliations:** 1 Department of Physiology, University of Hong Kong, Pokfulam, Hong Kong; 2 Department of Physiology, Chinese University of Hong Kong, Shatin, Hong Kong SAR, China

**Keywords:** Angiotensin IV, angiotensin IV receptor, chemoreceptor, type-I cells

## Abstract

Hypoxia regulates the local expression of angiotensin-generating system in the rat carotid body and the me-tabolite angiotensin IV (Ang IV) may be involved in the modulation of carotid body function. We tested the hypothesis that Ang IV-binding angiotensin AT_4_ receptors play a role in the adaptive change of the carotid body in hypoxia. The expression and localization of Ang IV-binding sites and AT_4_ receptors in the rat carotid bodies were studied with histochemistry. Specific fluorescein-labeled Ang IV binding sites and positive staining of AT_4_ immunoreactivity were mainly found in lobules in the carotid body. Double-labeling study showed the AT_4_ receptor was localized in glomus cells containing tyrosine hydroxylase, suggesting the expression in the chemosensitive cells. Intriguingly, the Ang IV-binding and AT_4_ immunoreactivity were more intense in the carotid body of chronically hypoxic (CH) rats (breathing 10% oxygen for 4 weeks) than the normoxic (Nx) control. Also, the protein level of AT_4_ receptor was doubled in the CH comparing with the Nx group, supporting an upregulation of the expression in hypoxia. To examine if Ang IV induces intracellular Ca^2+^ response in the carotid body, cytosolic calcium ([Ca^2+^]_i_) was measured by spectrofluorimetry in fura-2-loaded glomus cells dissociated from CH and Nx carotid bodies. Exogenous Ang IV elevated [Ca^2+^]_i_ in the glomus cells and the Ang IV response was significantly greater in the CH than the Nx group. Hence, hypoxia induces an upregulation of the expression of AT_4_ receptors in the glomus cells of the carotid body with an increase in the Ang IV-induced [Ca^2+^]i elevation. This may be an additional pathway enhancing the Ang II action for the activation of chemoreflex in the hypoxic response during chronic hypoxia.

## INTRODUCTION

The carotid body is the major peripheral organ responding to rapid changes in arterial chemical content such as partial pressure of oxygen and carbon dioxide, and plays important roles in the cardiopulmonary responses to hypoxia and hypercapnic acidosis. Type-I (glomus) cells in the carotid body are the chemosensitive cells because they are apposed to the nerve endings and are the main cell types containing catecholamines, presumably the source of dopamine release during sensory transduction of the physiological stimuli [1, 2]. Essentially, glomus cells elevates cytosolic calcium ([Ca^2+^]_i_) levels during hypoxia or hypercapnic acidosis [3–6]. The [Ca^2+^]_i_ elevation couples the stimuli to the vesicular secretion of the neurotransmitters including catecholamines, acetylcholine and ATP for the increase in afferent nerve activity that activates the central responses for the physiological changes to compensate metabolic needs.

The expression and localization of several key reninangiotensin system (RAS) components, notably angiotensiogen (AGT), which is an obligatory element for the existence of an intrinsic RAS have been demonstrated in the carotid body [7]. Protein and mRNA of AGT were specifically localized to the glomus cells. However, mRNA of renin was not detected while mRNA of angiotensin-converting enzyme (ACE) was found [7]. These results suggest that an intrinsic angiotensin-generating system is operating in the carotid body, probably *via* a locally renin-independent pathway. Hence, the locally produced angiotensin II (Ang II) could act *via* paracrine or autocrine on the angiotensin AT_1_ receptors expressed in the carotid body [8, 9].

Interestingly, chronic hypoxia causes an upregulation of the expression and localization of AGT mRNA and protein levels in the glomus cells of the rat carotid body [10]. In addition, mRNA expression of ACE was also increased by chronic hypoxia [10]. This is supported by studies with enzyme assay showing an increase in ACE activity in the chronically hypoxic rat carotid body comparing with the normoxic control [11]. These findings suggest that chronic hypoxia activates the local components of an intrinsic angiotensin-generating system in the rat carotid body.

In addition to Ang II and its ligand-binding receptors AT_1_ and AT_2_, recent studies have shown that a metabolite of Ang II, a pentapeptide containing the 3-8 fragment of Ang II, namely angiotensin IV (Ang IV), is a biologically active peptide of the RAS. It has also been demonstrated that high affinity binding sites for Ang IV, known as the angiotensin AT_4_ receptor, localized in various tissues such as the rat brain and kidney [12–14]. In the brain, Ang IV enhances the release of acetylcholine in hippocampal slices and improves memory in rats with dementia [15]. Recent studies identified AT_4_ receptor as insulin-regulated aminopeptidase which is a member of the M1 family of aminopeptidases [16]. The signaling cascades coupling to the ligand binding of the AT_4_ receptors are not clear and may involve multiple signaling pathways [14]. Recent evidence also suggests that an activation of AT_1_ receptors could be involved in the cellular effects of Ang IV on intracellular signaling events [17, 18]. At the moment, the expression of the AT_4_ receptor in the carotid body and its regulation by hypoxia remains unknown. In this study, we tested the hypothesis that AT_4_ receptors are present in the rat carotid body and play a role in the adaptive change in chronic hypoxia. In addition, we investigated the effect of Ang IV on the [Ca^2+^]_i_ level of the glomus cells because of the physiological significance of the [Ca^2+^]_i_ elevation in the chemotransduction.

## MATERIALS AND METHODOLOGY

### Preparation of Animals

The experimental protocol for this study was approved by the Committee on the Use of Live Animals in Teaching and Research of the University of Hong Kong and the Animal Ethical Committee of the Chinese University of Hong Kong. For the exposure of rats in normobaric hypoxia [19], one-month-old Sprague-Dawley rats were placed in a chamber made of Perspex. The chamber was continuously vented by pumping in room air (2 liter/min) and the oxygen fraction of the gas inside the chamber was kept at 10±0.5%. The oxygen level was continuously monitored by an oxygen analyser (Vacumed, CA, USA) which provided the servo-feedback signal for the control of solenoid values which gated the inflow of pure nitrogen. Animals were kept in the chamber for 4-5 weeks and they were freely accessible to water and chow. The humidity and carbon dioxide level was maintained by dehumidifier and soda lime. In every 2-3 days, the chamber was opened for 30 min for regular maintenance. For normoxic controls, litters were kept in the same room but were supplied with room air.

### Isolation of Carotid Body and Dissociation of Type-I Glomus Cells

Following deep anesthesia with halothane, rats were decapitated and the carotid bifurcation was excised rapidly. For the dissociation of glomus cells, the carotid body was carefully dissected free from the bifurcation in chilled rat Ringer oxygenated with 95% O2 and 5% CO_2_. The carotid body was then incubated in a tissue bath with collagenase (0.06%) and protease (0.02%) in oxygenated Ringer for 30 min at 35±1^°^C [8]. Following enzymatic treatment, the carotid body cells were dispersed by gently triturating with glass pipettes. Cells were incubated in 5 μM fura-2 acetoxymethyl ester (fura-2AM, Molecular Probes) for 30 min at room temperature. The cells were then centrifuged at 200 g for 5 min and prepared for the spectrofluorimetric measurement of [Ca^2+^]_i_. Glomus cells in clusters of 8-10 cells were studied and the morphological criteria for their confirmation as glomus cells was according to the methods used in previous reports [8, 19].

### Fluorescence-Labeled Binding Assay

Carotid bifurcations were excised from normoxic and hypoxic rats (n=8) under deep anesthesia. The bifurcations were rinsed in PBS and frozen in iso-pentane. Cryosections (8 μm) were cut on a Cryostat and fixed with freshly prepared 4% paraformaldehyde (PFA) for 30 min. Sections were washed with PBS at room temperature for 10 min and then fixed in 2% PFA for 10 min, followed by PBS rinse for 10 min. After air dry, sections were incubated for 30 min at room temperature with one of the following conditions, which were described in details in a previous report [20]: (1) 20 nM fluorescein isothiocyanate (FITC)-labeled Ang IV (Auspep, Australia) in 5 ml Ang IV incubation buffer, which contained 59.3μl of 50 μM plummers inhibitor (DL-2-2-Mercaptomethyl-3-guanidinoetyhiopropanoic acid (Calbio-chem, CA, USA)) and 17.2 μl of 11μM bestatin and 30 mg of 0.6% BSA in PBS; (2) pre-incubation with 10 μM non-labeled Ang IV (angiotensin II (3-8) (Auspep, Australia)) for 15 min followed by 5 mL Ang IV incubation buffer with 20 nM FITC-labeled Ang IV; (3) 20 nM fluorescein isothiocyanate (FITC)-labeled Ang IV in 5 ml Ang IV incubation buffer with 4.8 μl of 1mg/ml (1 μM) Sarile ([Sar^1^, Ile^8^]-angiotensin II; Sar-Arg-Val-Tyr-Ile-His-Pro-Ile). Following incubation, slides were washed twice in PBS for 1 min and then by double distilled water for 30 min at 4°C. Sections were dry overnight and examined with a fluorescent microscope with a DC 200 digital camera (Leica Microsystems Ltd., Heerbrugg, Switzerland). Non-specific bindings were assessed in sections with the non-labeled Ang IV and the specific bindings of Ang IV were demonstrated in sections with Sarile that is not readily metabolized to Ang III and Ang IV.

### Immunohistochemistry

Cryosections were then processed for indirect immunofluorescent double staining as reported previously [7]. Briefly, sections were incubated overnight at 4°C with rabbit anti-rat AT_4_ receptor serum, diluted to 1:1000, followed with anti-tyrosine hydroxylase serum (Chemicon International, USA), diluted to 1:300 overnight at 4°C. After rinses in PBS, the primary antibodies were detected using anti-rabbit serum labeled with rhodamine (1:100, Jackson, USA) for AT_4_ receptor and anti-sheep serum labeled with AMCA (1:100, Jackson, USA) for TH at room temperature for 2 h. Positive immunoreactivity for AT_4_ receptor (red) and for TH (blue) was examined with a fluorescent microscope with a DC 200 digital camera (Leica Microsystems Ltd., Heerbrugg, Switzerland). For the control, primary antibodies were substituted with buffer and sections were incubated with rabbit nonimmune serum.

### Western Blot Analysis

The procedures were reported previously [7]. Carotid bodies were dissected free and frozen at - 70 ° C. Carotid bodies (n = 20) were homogenized at 4 °C in water (1:9, w / v) containing 10 mM EDTA and 1 mM phenylmethylsulphonyl fluoride. The protein from resultant supernatant was determined (Bio-Rad Protein Assay) for sodium dodecy sulphate-polyacrylamide gel electrophoresis (SDS-PAGE). Proteins (5 - 10 mg / lane) were subjected to electrophoresis on a 12 % (w / v) polyacrylamide gel in SDS, and the gel was subsequently processed for electroblotting to polyvinylidene difluoride (PVDF) membrane. The blotted PVDF membrane was saturated with 5 % (w / v) of skimmed milk in PBS, pH 7.4 and 0.1 % (w / v) of Tween 20 for 1 h at room temperature. The membrane was sequentially incubated in rabbit anti-rat AT_4_ receptor serum (1 : 2500) overnight at 4°C and a horse-radish peroxidase-labeled anti-rabbit IgG (1 : 200 dilution, Boehringer Mannheim, Germany) for 2 h at room temperature. After thorough washing, the positive band was revealed using ECL western blotting detection reagents and autoradiography film (Amersham, Biosciences, UK). The florescence intensity of the bands was quantified using an IMAGE QUANT software (Molecular Dynamics, USA).

### Spectrofluorimetric Measurement

[Ca^2+^]_i_ was measured in fura-2 loaded glomus cells freshly dissociated from rat carotid bodies as described in previous studies [8, 19]. The cells were seeded on a cover slip placed in the stage of an inverted microscope. The microscope was coupled with a dual-wavelength excitation spectrofluorimeter (Photon Technology International, NJ, USA). The cells were perfused with HEPES buffer at 0.5 ml/min at room temperature (∼22°C). The fluorescence intensity in the background was measured and subtracted from the signals. Fluorescent signals were obtained at 340 and 380 nm excitation wavelengths. The ratio of the fluorescence intensity (340/380 nm) was used to estimate [Ca^2+^]_i_ in the glomus cells. The [Ca^2+^]_i_ was calculated by using the equation:

$${{\text{[Ca}}^{\text{2+}}\text{]}}_{\text{i}}{\text{ = K}}_{\text{d}}{\text{ [(R}}_{\text{o}}{\text{ - R}}_{\text{min}}{\text{)/(R}}_{\text{max}}{\text{ - R}}_{\text{o}}\text{)] β}$$

where R_o_ is the fluorescence ratio, Rmin is the fluorescence ratio at zero Ca^2+^, R_max_ is the fluorescence ratio at saturated Ca^2+^, K_d_ is the dissociation constant for fura-2 (224 nM) and β is the ratio of 380 nm fluorescence intensity at zero Ca^2+^ to 380 nm fluorescence intensity at saturated Ca^2+^.

### Experimental Paradigm

Dosage dependence was determined by the [Ca^2+^]i response to Ang IV at 0.01, 0.1, 1 and 10 μm. Ang IV was administered directly into the 0.5 ml chamber with a fine pipette without disrupting the fluid in the chamber. At the end of the experiment, acute hypoxia was induced by sodium cyanide (NaCN, 1 mM, in bolus) to confirm the chemosensitivity of the glomus cells.

### Materials and Pharmacological Agents

The rat Ringer solution contained (mM): NaCl 125, KCl 3.1, NaHCO_3_ 26, NaH_2_PO_4_ 1.25, MgSO_4_ 1.3, CaCl_2_ 2.4, D-Dextrose 10. The HEPES Ringer contained (mM): NaCl 140, KCl 3, NaH_2_PO_4_ 1.25, MgCl_2_ 1, CaCl_2_ 1, HEPES 10, D-Dextrose 25 (pH = 7.35-7.4). Losartan (1 μM) an antagonist for AT_1_ receptors was dissolved in HEPES Ringer solution. The concentrations of Ang IV and NaCN were 5 mM and 1 M, respectively, for the injection. Fura-2AM was dissolved in dimethyl sulfoxide (DMSO) in 1 mM stock. Drugs were purchased from Sigma or mentioned otherwise.

### Statistical Analysis

All gel images from Western blot study were analyzed and quantified by an UV illuminator (FluroChem 8000 Advanced Fluorescence, Chemiluminescence and Visible Light Imaging, Alpha Innotech Corporation, CA, USA). For the [Ca^2+^]_i_, the resting and peak values of the fluorescence ratio of 340/380 nm or calibrated [Ca^2+^]_i_ (nM) of the responses during drug treatment were calculated. Values were normalized to% control if needed and were presented as mean ± standard error. Statistical comparisons were made with the unpaired *t*-test for comparing the normoxic control and hypoxic group. ANOVA with *post hoc* test (Dunnett’s *t*-test) were used for multiple comparisons of values in drugs studies among groups with different doses. Differences were considered significant at *p*<0.05.

## RESULTS

**Fig. (1):**
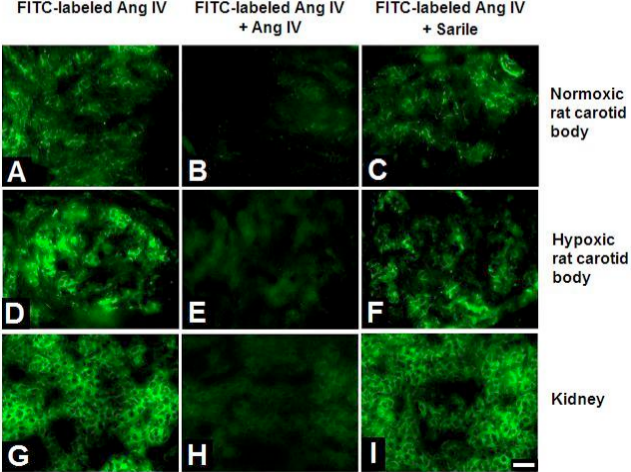
Angiotensin IV (Ang IV)-binding sites in normoxic (Nx) and chronically hypoxic (CH) rat carotid body. Panels on top show FITC-coupled Ang IV binding (**A**), + Ang IV (**B**), + Sarile (**C**) in Nx group. Panels in middle show FITC-coupled Ang IV binding (**D**), + Ang IV (**E**), + Sarile (**F**) in CH group. Panels at bottom show FITC-coupled Ang IV binding (**G**), + Ang IV (**H**), + Sarile (**I**) in rat kidney, which was used as the positive control. Concentration of FITC-coupled Ang IV, unlabeled Ang IV and Sarile were 0.2, 10, 1 μM, respectively. Calibration bar is 20 μm.

Specific bindings of FITC-labeled Ang IV were obtained in the carotid body and also in the kidney served as a positive control. Fig. (1) shows the Ang IV-binding sites in the Nx (Fig. 1**A**) and CH (Fig. 1**D**) carotid body and in the kidney (Fig. 1**G**). The pattern of the labeling illustrates that the binding sites were localized in glomerular clusters in the carotid bodies, suggesting the expression in the chemosensitive glomus cells. In addition, the intensity of the fluorescence labeling was more intense in the CH than that of the Nx group. The binding was abolished by prior incubation of the sections with Ang IV without FITC labeling (Fig. 1**B**,**E**,**H**). Also, the FITC-labeled Ang IV binding was not affected by the addition of excessive amount of Sarile (Fig. 1**C**,**F**,**I**), which saturated the AT_1_ and AT_2_ binding sites.

**Fig. (2):**
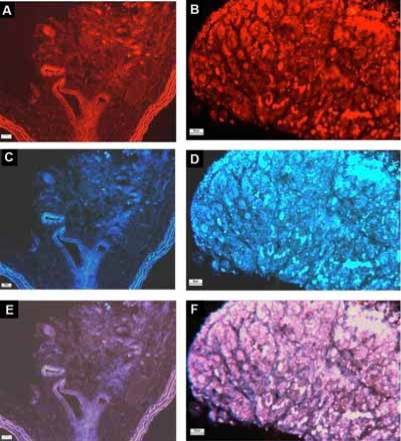
The expression and localization of AT_4_ receptor-immunoreactivity in (**A**) Nx and (**B**) CH rat carotid body. Note the intensity of staining is at a high level in the CH comparing with that of the Nx group. Sections with tyrosine hydroxylase (TH) for the Nx and CH carotid bodies are shown in **C** and **D**. **E** and **F** are overlays of the AT_4_ receptor- and TH-stainings. Calibration bars are 20 and 50 μm, respectively, for low (**A**, **C**, **E**) and high magnification (**B**, **D**, **F**).

The localization of protein expression of the AT_4_ receptors in the carotid body is shown in Fig. (2). Positive staining of AT_4_ receptor-immunoreactivity was obtained in Nx and CH carotid bodies (Fig. 2**C**,**D**). The immunostaining was localized in lobules of the carotid body, which was also positively stained with TH-immunoreactivity (Fig. 2**A**,**B**), confirming that the AT_4_ receptor expression was in the chemosensitive glomus cell. Additionally, the intensity of the staining was at a high level in the CH comparing with that of the Nx group, indicating an increase in AT_4_ receptor expression in the CH carotid body.

**Fig. (3):**
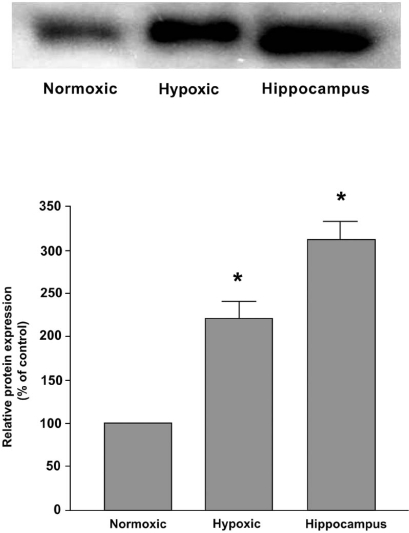
Protein expression of AT_4_ receptors in Nx and CH rat carotid body. Note the level of protein expression increased significantly in the CH group. The expression in the hippocampus serves as the positive control. Densitometric analysis depicts the relative expression of AT_4_ receptor protein in the CH group of carotid bodies and the expression is normalized as percent of the Nx group. Statistical significance (n=5, p<0.05, unpaired-*t* test) is shown in asterisk when comparing with the Nx group.

To examine the protein expression of AT_4_ receptor in the Nx and CH rat carotid bodies, Western blot analysis was performed and results are shown in Fig. (3). The level of protein expression increased significantly (p<0.05) by about two folds in the CH comparing with the Nx group. The AT_4_ receptor was also expressed in the hippocampus served as the positive control.

**Fig. (4):**
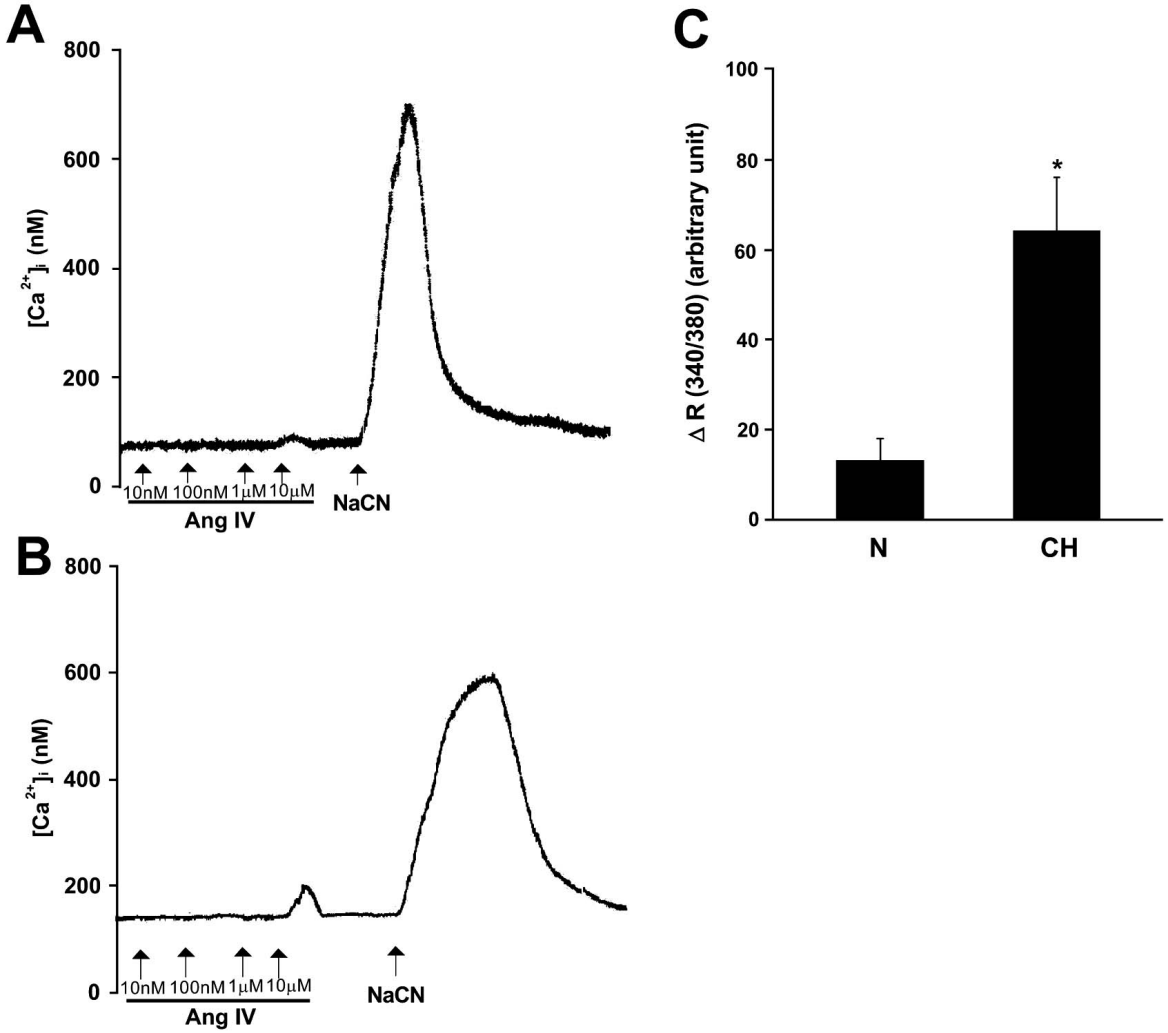
The [Ca^2+^]_i_ response to exogenous Ang IV in dissociated glomus cells of the rat carotid body from Nx control (**A**) and CH group (**B**). The records were made in cluster of 8-10 cells loaded with fura-2. Ang IV-induced [Ca^2+^]_i_ elevation was more significant in the CH glomus cells than that of the Nx group. Note that the transient [Ca^2+^]_i_ increase following the injection of Ang IV (0.01-10 μM) into the bath. In addition, histotoxic hypoxia with sodium cyanide (NaCN, 1 mM) increased the [Ca^2+^]i of the cells. Results are summarized in (**C**). On average, the [Ca^2+^]_i_ elevation (ΔR (340/380)) induced by 10 μM Ang IV in the CH group (n=9) was significantly greater than that of the Nx control (n=7). Statistical significance (p<0.05, unpaired-*t* test) is shown in asterisk when comparing with the other groups.

To investigate whether Ang IV stimulates intracellular Ca^2+^ response in the carotid body, the [Ca^2+^]_i_ level was measured by spectrofluorimetry in fura-2-loaded glomus cells dissociated from the carotid bodies. Exogenous Ang IV (10 μM) elevated [Ca^2+^]_i_ in clusters of glomus cells, although 0.1-1 μM Ang IV had only negligible effects on the [Ca^2+^]_i_ levels (Fig. 4). The peak [Ca^2+^]_i_ level was reached shortly following Ang IV stimulation and the [Ca^2+^]_i_ levels gradually returned to the resting level within 3 minutes. In the CH glomus cells, the peak [Ca^2+^]_i_ response to Ang IV was significantly greater that that of the Nx group (Fig. 4). On average, the levels of [Ca^2+^]_i_ elevation induced by Ang IV (10 μM) were 51.0 ± 10.1 nM (n=9) and 13.4 ± 4.6 nM (n=7), respectively, in the CH and Nx group. Histotoxic hypoxia with sodium cyanide (1 mM) increased the [Ca^2+^]_i_ level in all of the glomus cells. Results suggest that Ang IV can activate the [Ca^2+^]_i_ response in the chemosensitive glomus cells and that the Ang IV-induced [Ca^2+^]_i_ response is enhanced by CH.

## DISCUSSION

This is the first study to demonstrate an expression of AT_4_ receptors in the rat carotid body and its upregulation in chronic hypoxia. Hence, using FITC-labeled Ang IV, a low intensity of binding is present in the rat carotid body. This is also supported by the positive staining of immunoreactivity against the AT_4_ receptor, which is co-localized with the tyrosine hydroxylase (TH)-positive glomus cell in the carotid body. In fact, the results of the FITC study are consistent with the immunohistochemical findings. The FITC-labeled cells were found in glomerular clusters of the carotid body, which is a main histological feature of the glomus cells. These lobules of chemosensitive cells are positive with the immunoreactivity of AT_4_ receptor, which contain TH for the synthesis of catecholamines as one of the neurotransmitters released during chemotransduction. Ang IV is a metabolite of Ang II and binds to distinct AT_4_ receptors that are localized in various tissues such as the brain and kidney [12–14]. In the carotid body, the Ang IV-binding of the AT_4_ receptor is highly specific because the fluorescein-coupled Ang IV binding was not displaced by Sarile, which is a ligand of the AT_1_ and AT_2_ receptors, whereas displaced by the presence of excess Ang IV. In addition, distinct AT_4_ receptors are localized in TH-positive glomus cells, implying a functional role in the modulation of the activity of the glomus cells. This may enhance the excitatory effect of Ang II on the carotid chemoreceptor mediated by the AT_1_ receptors that are also expressed in the glomus cells.

Our results suggest an upregulation of the expression of AT_4_ receptors in the carotid body in chronic hypoxia. Hence, we found an increased intensity of staining of the Ang IV-binding sites and AT_4_ receptors in the CH when comparing with that of the Nx carotid bodies. Quantitatively, the expression of AT_4_ receptors had a 2-fold increase in the CH carotid bodies comparing with the Nx control. Thus the AT_4_ receptor could be complementary to the RAS component in the carotid body, because of its upregulation by chronic hypoxia. Previous studies have shown that chronic hypoxia increases chemoreceptor sensitivity to Ang II in the rat carotid body [19, 21]. The augmentation of Ang II sensitivity is mediated by an upregulation of AT_1_ receptors in the glomus cells of the carotid body [19, 21]. These results are consistent with the idea that an upregulation of the RAS in the carotid body plays a prominent role in the local adaptive response to hypoxia.

Ang IV and the AT_4_ receptors have been reported to be involved in diverse functions, including blood flow regulation [22, 23], learning and memory function [24, 25], cell growth [26–28] and anti-apoptosis [29]. We found that Ang IV can induce a significantly greater [Ca^2+^]_i_ elevation in the CH glomus cells than that of the Nx control, supporting the idea that the upregulation of the AT_4_ receptor plays a functional role in the carotid response to chronic hypoxia. In this context, Lochard *et al*. [17] reported that Ang IV can mobilize [Ca^2+^]_i_ *via* AT_1_ receptors mediated by an allosteric mechanism and that AT_1_ receptor antagonist can block the hypertension in transgenic mice with Ang IV release in the brain. Also, it has been shown that Ang IV stimulates tyrosine kinases activities *via* an AT_1_ receptor pathway in the rat pituitary tumor cells [18]. Furthermore, it is likely that multiple pathways and intracellular signals mediate the cellular effects of AT_4_ receptors on the carotid chemosensitive cells. It has been proposed that Ang IV (1) can act as potent inhibitor of insulin-regulated aminopeptidase, which may prolong the action of endogenous promnestic peptides; (2) may modulate glucose uptake by modulating trafficking of glucose transporters; (3) may transduce the signal initiated by ligand binding to its C-terminal domain to the intracellular domain that interacts with several cytoplasmic proteins [14]. Hence, activation of AT_4_ receptors in the glomus cells may interact with the AT_1_ receptor and its signalling pathway, and so this may activate protein kinase C for the Ang IV-induced [Ca^2+^]_i_ response. Although we can not rule out the possibility that Ang IV may directly activate the AT_1_ receptor with a dosage of exogenous Ang IV in the micromolar range, our binding studies with Sarile did not support the interpretation of such a non-specific binding of Ang IV to the AT_1_ receptor. Nevertheless, the functional significance of the AT_4_ receptor expression may not be fully reflected by the [Ca^2+^]_i_ response induced by Ang IV and the details of the Ang IV action and possible interaction with the AT_1_ receptors and its signaling cascades await elucidation by further studies. Also, potential roles of Ang IV in heart failure and other cardiovascular diseases remain to be investigated with different animal models or experimental settings, which can not be closely mimicked by chronic hypoxia.

AT_4_ receptors could play a more prominent role following its upregulation in chronic hypoxia. This is in parallel to the fact that the expression and function of the local RAS in the carotid body is also upregulated in chronic hypoxia [9]. Presumably, this can increase the amount of Ang II locally produced in the carotid body, which in turn causes an elevated level of Ang IV for the activation of intracellular signaling cascade leading to the [Ca^2+^]_i_ elevation. Moreover, circulating RAS is stimulated by acute hypoxia [30, 31] and chronic hypoxia [32, 33]. The plasma Ang II level increases during hypoxic conditions [34] and peripheral infusion of Ang II stimulates ventilation [35, 36]. Thus, the functional AT_4_ receptor in the carotid body may be an additional pathway enhancing the Ang II action for the activation of chemoreflex and sympathetic activity as well as functional changes such as in the hypoxic sensory response during prolonged hypoxia. Also, through unknown mechanisms possibly involving the carotid chemoreceptor, chronic hypoxia causes diuresis and sodium depletion; hence enhances the physiological responses to retain salt and water for the fluid balance [37]. The activation of AT_4_ receptors in the carotid body may be an additional pathway enhancing the Ang II action for the activation of chemoreflex in the physiological response during chronic hypoxia.
